# An inference method from multi-layered structure of biomedical data

**DOI:** 10.1186/s12911-017-0450-4

**Published:** 2017-05-18

**Authors:** Myungjun Kim, Yonghyun Nam, Hyunjung Shin

**Affiliations:** Department of Industrial Engineering, Ajou University, 206 Worldcup-ro, Yeongtong-gu, Suwon 16499 South Korea

**Keywords:** Integrative inference on biomedical data, Semi-supervised learning, Semi-supervised learning for multiple networks, Symptom-disease multi-layered network, Disease co-occurrence prediction

## Abstract

**Background:**

Biological system is a multi-layered structure of omics with genome, epigenome, transcriptome, metabolome, proteome, etc., and can be further stretched to clinical/medical layers such as diseasome, drugs, and symptoms. One advantage of omics is that we can figure out an unknown component or its trait by inferring from known omics components. The component can be inferred by the ones in the same level of omics or the ones in different levels.

**Methods:**

To implement the inference process, an algorithm that can be applied to the multi-layered complex system is required. In this study, we develop a semi-supervised learning algorithm that can be applied to the multi-layered complex system. In order to verify the validity of the inference, it was applied to the prediction problem of disease co-occurrence with a two-layered network composed of symptom-layer and disease-layer.

**Results:**

The symptom-disease layered network obtained a fairly high value of AUC, 0.74, which is regarded as noticeable improvement when comparing 0.59 AUC of single-layered disease network. If further stretched to whole layered structure of omics, the proposed method is expected to produce more promising results.

**Conclusion:**

This research has novelty in that it is a new integrative algorithm that incorporates the vertical structure of omics data, on contrary to other existing methods that integrate the data in parallel fashion. The results can provide enhanced guideline for disease co-occurrence prediction, thereby serve as a valuable tool for inference process of multi-layered biological system.

## Background

Omics is a comprehensive study of a specific layer in a cellular system [1] and the molecular components in each layer constitute the biological system. These layers include genome, epigenome, transcriptome, metabolome, proteome, etc., and can further be extended to clinical/medical layers such as diseasome, drugs, and symptoms. There exist complex interactions between each layers, such as translation, transcription, and reactions, and such interactions allow us to view biological system as a multi-layered structure of omics. In recent years, there has been great advances in high throughput experimental techniques and brought influx of omics data including DNA sequence data, mRNA, miRNA, methylation patterns, etc [2]. While there had been many works concerning single layer of omics data, complex interactions between different layers hinder one from capturing comprehensive information on total system. Therefore, comprehensive analysis of multiple omics is required for more profound understanding of the total biological system [3]. One integrative approach for multiple levels of information that is receiving much attention is network-based or graph-based approach. A network or a graph concerning omics data consists of nodes and edges, where nodes represent biological components, such as genes or diseases, and edges represent relationships or interactions among them [4]. The main reason for the popularity of network-based analysis of biological system lies on the fact that the network structure can captures associations of biological components while managing large amount of data [5]. The network can vary from gene co-expression networks [6–9], protein networks [10–13], metabolic networks [14, 15], disease networks [16, 17], and many more, for single layered networks while multi-layered networks can be created by connecting the layers using data that reflects interactions between different layers [18].

Given a multi-layered network, one can extend the usage of such networks by implementing prediction process for finding traits (or labels) of interest with machine learning algorithms. While many traits have been discovered in numerous studies, there still remain a large room for finding more unknown traits of biological components. Instead of leaving unknown components in dark space, one can utilize both known and unknown components with semi-supervised learning. Semi-supervised learning (SSL), in general, deals with both labeled and unlabeled data where labeled data are given scarcely compared to vast amount of unlabeled data, and obtaining labels for unknown traits is costly. In this sense, SSL can serve as a cost-effective tool for prediction process [19]. For SSL in network setting [20–24], the key idea is the ‘label propagation’ [25] where known labels propagate to neighboring unlabeled data points through edges. Through label propagation and basic kernel of graphs using graph Laplacian [26], we obtain predictive values for unlabeled data, which we can utilize for prediction process for networks of biological systems.

In past works, there have been extensive studies incorporating SSL for various omics data. In [27–29] graph integration method, consisting of finding convex combination of graph Laplacians, is applied to four different types of yeast protein networks along with SSL to predict protein functions and also extends to protein function prediction by incorporating deletion process of noisy connections [30]. For more practical purpose on clinical data, [31–33] applies graph integration methods on multiple graphs from CNA, methylation, miRNA, and gene expression along with SSL to predict clinical outcomes of cancer. In [34], SSL schemes are applied to predict disease genes from protein-protein interaction network, constructed with multiple proteomics and genomic data. In [35], SSL was applied to predict synthetic genetic interactions from integrated network of protein-protein interaction, protein complex, and gene expression data. For inter-layer relationships, [36] provides algorithms for reconstructing intra-layer relations by utilizing SSL and inter-layer relations between different levels of genomic data. In [37], the authors provides miRNA-disease associations by utilizing SSL algorithm. In [38], SSL was applied to for disease comorbidity scoring for complemented disease network of metabolic disease group.

Most of the above works, however, only consider integrating multiple sources of data in parallel fashion, ignoring hierarchical, or vertical structure of multi-omics data. Furthermore, only few machine learning algorithms, including SSL, deals with networks of vertical structure. The purpose of the paper is to develop a semi-supervised learning algorithm for multiple layered networks that utilize matrix separation and graph integration method in vertical fashion. For biological systems, however, vast number of components in each layers and countless unknown relations between different layers cause issues of computational complexity and sparseness for analyzing with multi-layered networks. To alleviate the problems, we propose an efficient matrix inversion algorithm composed with Nyström method [39] and Woodbury formula [40]. The remainder of the paper is organized as the following. In Methods, we discuss graph based semi-supervised learning for multiple-layered networks. In Experiments and Results and Discussion, we present experimental results of the proposed algorithm that was applied to disease co-occurrence prediction problem on two layered network of symptom and disease.

## Methods

### Graph based semi-supervised learning

In graph based semi-supervised learning, a set of data can be represented by a graph *G*(*V, E*) which consists of nodes (*V*) and edges (*E*). Given a graph *G*(*V, E*) for *n* data points, nodes represent data points with *V* = {*x*_1_, *x*_2_, …, *x*_*n*__*tween dats epresetn* }. and edges represent similarities between data points. The similarities are given by the weight matrix *W*, where elements, *W*_*ij*_, of *W* represent strength of connection between nodes *x*_*i*_ and *x*_*j*_. The problem of semi-supervised learning on graph *G*(*V, E*) deals with labeled and unlabeled nodes where labeling is given by *Y* = {*Y*_*l*_, *Y*_*u*_} with *Y*_*l*_ ∈ {−1, 1} for labeled nodes and *Y*_*u*_ = 0 for unlabeled nodes. Through learning process, we determine the output vector *f* = (*f*_1_, *f*_2_, …, *f*_*n*_)^*T*^ using available information and minimizing the following quadratic cost functional [41]:

1 $$ \begin{array}{c}\hfill \mathrm{minimize}\hfill \\ {}\hfill f\hfill \end{array}\ {\displaystyle \sum_i^n}{\left({f}_i-{Y}_i\right)}^2+\mu {\displaystyle \sum_{i, j}^n}{W}_{i j}{\left({f}_i-{f}_j\right)}^2. $$

By the symmetry of the weight matrix, problem (1) can be translated into

2 $$ \begin{array}{c}\hfill \mathrm{minimize}\hfill \\ {}\hfill f\hfill \end{array}\ {\left( f- Y\right)}^T\left( f- Y\right)+\mu {f}^T L f, $$

where *L* is the graph Laplacian [26] defined as *D–W* for *D* = *diag*(*d*_*i*_) and *d*_*i*_ = ∑_*j*_ *W*_*ij*_. In (2), the first term is the loss term for consistency with initial labeling, the second term is the smoothness term for consistency with geometry of the data, and μ is a parameter for trade-off between the loss term and the smoothness term [41]. The solution to minimization problem (2) is given by:

3 $$ f={\left( I+\mu L\right)}^{-1} Y, $$

where I is the identity matrix.

### Semi-supervised learning for multi-layered biomedical data

**Fig. 1:**
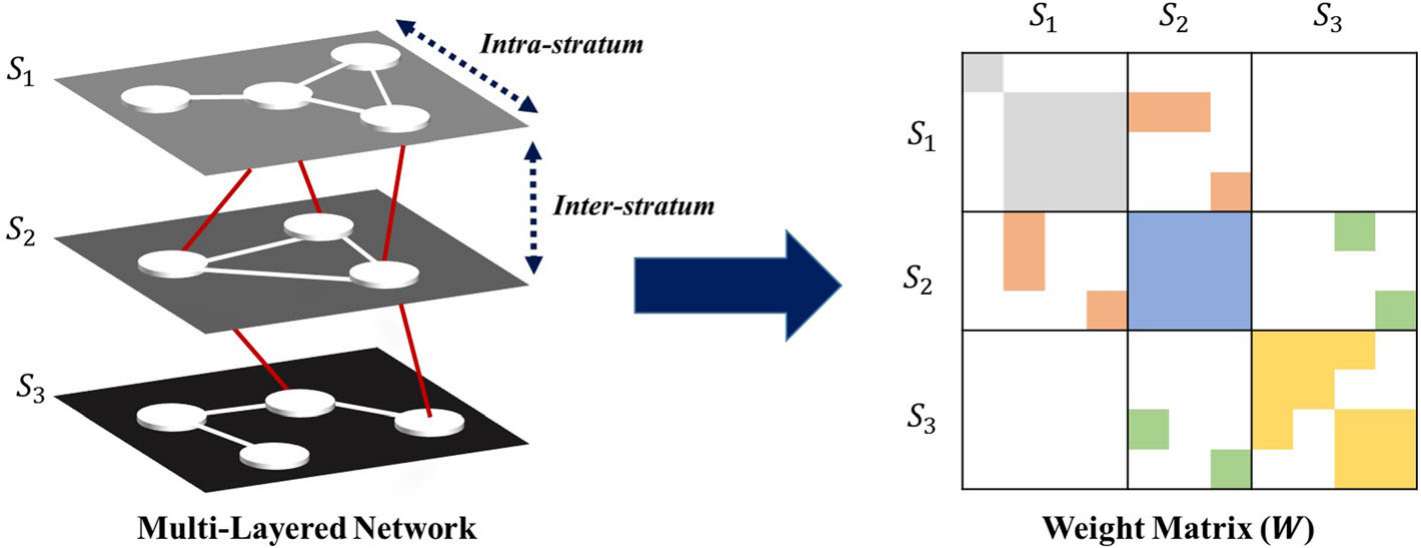
Multi-layered graph for three layers with block tri-diagonal structure of its weight matrix

For multi-layered biomedical data, it can be represented by multi-layered graph, *G*(*V, E, S*), which consists of nodes (*V*), edges (*E*), and strata (*S*). In addition to nodes and edges, strata in *G*(*V, E, S*) denote *K* distinct layers with *S* = {*S*_1_, *S*_2_, …, *S*_*K*_}. Each *G*(*V, E, S*) contains intra- and inter-layer relations, where the former characterize relations between two nodes in same layer and the latter characterize relations between two nodes each of which belongs to different adjacent layer. Given a graph *G*(*V, E, S*) with *K* number of layers and *n*_*k*_ data points for each layer *k*, the weight matrix *W* is a *N* × *N*, where *N* = *n*_1_ + *n*_2_ + … + *n*_*K*_, block tri-diagonal matrix with 3*K* − 2 non-zero blocks. *K* symmetric diagonal blocks represent intra-layer relations and 2*K* − 2 rectangular banded diagonal blocks represent inter-layer relations. Figure 1 depicts a multi-layered graph for three layers with structure of its corresponding weight matrix. An exemplary network would be a multi-layered network with *S*_1_, *S*_2_, and *S*_3_ as symptoms, diseases, and proteins, respectively, in the context of disease co-occurrence prediction. To incorporate graph based semi-supervised learning into multi-layered omics systems, we first apply matrix separation on the weight matrix, *W*, then implement graph integration method [28].

First, matrix separation is a representation of a block matrix with summation of its sub-matrices of same dimension with associated blocks. For the weight matrix *W* in a multi-layered graph, let ${W}^{\left\{{S}_p,\ {S}_q\right\}}$ be a matrix that only contains a sub-block of *W* associated with stratum *S*_*p*_ and *S*_*q*_, masking other blocks to zeros. Then, we have

4 $$ W={\displaystyle \sum_{S_p,{S}_q}^K}{W}^{\left\{{S}_p,\ {S}_q\right\}}, $$

where *S*_*p*_ = *S*_*q*_ denotes a sub-matrix for intra-layer relation of *S*_*p*_ (or *S*_*q*_) and *S*_*p*_ ≠ *S*_*q*_ denotes a sub-matrix for inter-stratum relation of two different strata, *S*_*p*_ and *S*_*q*_. Since effects of label propagation can be different for intra-layer and inter-layer connections, we want to look at them separately. Using (4), we have

5 $$ W={\displaystyle \sum_{S_p,{S}_q}^K}{W}^{\left\{{S}_p,\ {S}_q\right\}}={\displaystyle \sum_{S_p={S}_q}^K}{W}^{\left\{{S}_p,\ {S}_q\right\}}+{\displaystyle \sum_{S_p\ne {S}_q}^K}{W}^{\left\{{S}_p,\ {S}_q\right\}}\equiv {W}^{\left\{ intra\right\}}+{W}^{\left\{ inter\right\}}, $$

where *W* ^{*intra*}^ consists of *K* diagonal blocks of intra-layer relations and *W* ^{*inter*}^ consists of 2*K* − 2 banded diagonal blocks of inter-layer relations. By accounting for different parameters *μ*_*a*_(≥0) and *μ*_*b*_(≥0) for *W* ^{*intra*}^ and *W* ^{*inter*}^, respectively, the formalization (1) becomes

6 $$ \begin{array}{c}\hfill \mathrm{minimize}\hfill \\ {}\hfill f\hfill \end{array}\ {\displaystyle \sum_i^n}{\left({f}_i-{Y}_i\right)}^2+{\mu}_a{\displaystyle \sum_{i, j}^n}{W}_{i j}^{\left\{ i ntra\right\}}{\left({f}_i-{f}_j\right)}^2+{\mu}_b{\displaystyle \sum_{i, j}^n}{W}_{i j}^{\left\{ i nter\right\}}{\left({f}_i-{f}_j\right)}^2. $$

Since *W* ^{*intra*}^ and *W* ^{*inter*}^ themselves are weight matrices, each has graph Laplacian denoted as *L* ^{*intra*}^ and *L* ^{*inter*}^, respectively. This implies that we can translate problem (5) into

7 $$ \begin{array}{c}\hfill \mathrm{minimize}\hfill \\ {}\hfill f\hfill \end{array}\ {\left( f- Y\right)}^T\left( f- Y\right)+{f}^T\left({\mu}_a{L}^{\left\{ intra\right\}}+{\mu}_b{L}^{\left\{ inter\right\}}\right) f. $$

As sum of positive semidefinite matrices is still positive semidefinite, *μ*_*a*_ *L* ^{*intra*}^ + *μ*_*b*_ *L* ^{*inter*}^ *s* is positive semidefinite. This means that the optimization problem (6) is a convex problem, where the solution is given as

8 $$ f={\left( I+{\mu}_a{L}^{\left\{ intra\right\}}+{\mu}_b{L}^{\left\{ inter\right\}}\right)}^{-1} Y. $$

Note that when *μ*_*b*_ = 0, (7) reduces to (3).

### Revised matrix inversion method for multi-layered biomedical data

In eq. (7), the matrix inversion requires *O*(*N* ^3^) computational complexity for *N* number of data. For multi-layered structure of omics, the size of data can be tremendous which implies expensive computation for (7). To overcome such difficulty, various inversion algorithms for block tri-diagonal matrices, such in [42–45], can be considered. These algorithms, however, require square banded diagonal blocks which is not applicable since non-zero blocks in ${W}^{\left\{{S}_p,\ {S}_q\right\}}$ can be rectangular because of difference in sizes of different omics (*n*_*p*_ ≠ *n*_*q*_). In addition, sparseness of multi-layered structure of omics and the block tri-diagonal matrix can lead to inefficiency in matrix inversion involved in (7).

Revised matrix inversion method involves combination of Nyström method [39] and Woodbury formula [40]. The idea is to apply low rank approximation to *L* ^{*inter*}^ with Nyström method and utilize Woodbury formula to obtain the solution to problem (6). First, let us look at Nyström method and Woodbury formula.

[Nyström method] Nyström method is a low rank approximation of a positive semidefinite matrix from a subset of its columns. Given a positive semidefinite matrix *H* of size *n*, randomly sample *r* ≪ *n* columns, namely *C*. By defining *Q* as the intersection of *C* and its corresponding rows in *H*, Nyström approximation *Ĥ*, is given by

9 $$ H\approx \widehat{H}= C{Q}^{+}{C}^T, $$

where *Q* ^+^ is the pseudo-inverse of *Q* with rank of *Ĥ* equal to *r*.

[Woodbury formula] Woodbury formula matrix is inversion identity for sum of two matrices. Suppose *A* is an *n* × *n* invertible matrix, *B* is a *r* × *r* (*r* not necessarily equal to *n*) invertible matrix, *U* is a *n* × *r* matrix. Suppose furthermore that B^− 1^ + *U* ^*T*^ *A* ^− 1^ *U* is invertible. Then,

10 $$ {\left( A+ UB{U}^T\right)}^{-1}={A}^{-1}-{A}^{-1} U{\left({B}^{-1}+{U}^T{A}^{-1} U\right)}^{-1}{U}^T{A}^{-1}. $$

Woodbury formula is useful when computational cost of obtaining *A* ^− 1^ is cheap and the total matrix has sparse structure [43].

In eq. (7), *L* ^{*inter*}^ is a positive semidefinite matrix by the property of graph Laplacian [26], and thus applicable for Nyström method. By applying Nyström method to *L* ^{*inter*}^, we obtain

11 $$ {L}^{(inter)}\approx C{Q}^{+}{C}^T, $$

where *C* is a *n* × *r* (*r* ≪ *n*) matrix and *Q* ^+^ is a *r* × *r* matrix. Substituting the result to eq. (7) yields

12 $$ f={\left( I+{\mu}_a{L}^{\left\{ intra\right\}}+{\mu}_b C{Q}^{+}{C}^T\right)}^{-1} Y. $$

To use Woodbury formula, let *A* = *I* + *μ*_*a*_ *L* ^{*intra*}^, and *B* = *μ*_*b*_ *Q* ^+^, By Woodbury formula, we have the final solution to problem (6) in the form

13 $$ f={A}^{-1} Y-{A}^{-1} C{\left({B}^{-1}+{C}^T{A}^{-1} C\right)}^{-1}{C}^T{A}^{-1}{Y}^{-1}. $$

### Overview of the proposed method

The justification for using the proposed method starts with observing Woodbury formula used for matrix inversion in (11). From (11), we see that the matrix *A*, defined as *I* + *μ*_*a*_ *L* ^{*intra*}^, is a block diagonal matrix and that the total matrix has sparse structure arising from the property of block tri-diagonal matrix. Since obtaining the inverse of block diagonal matrix is cheap and the total matrix is sparse, we can infer from [43] that Woodbury formula is an effective approach for obtaining the inverse in eq. (11). The complexity for Woodbury formula (in fact the overall complexity) is given by

14 $$ O\left({\left( \max \kern0.5em \left\{{n}_1,\kern0.5em {n}_2,\dots, {n}_K\right\}\right)}^3+ r{N}^2\right), $$

where *n*_*k*_ denotes size of stratum *S*_*k*_ and *r* ≪ *N*.

In regards to Nyström method, a natural question could be brought upon selection of *L* ^{*inter*}^ for low-rank approximation. It is true that we could apply Nyström method on *μ*_*a*_ *L* ^{*intra*}^ + *μ*_*b*_ *L* ^{*inter*}^ as the sum of positive semi-definite matrices is still positive semidefinite. This approach, however, could lead to loss of structure and properties of each layer since we are approximating the graph Laplacian with randomly sampled columns. By selecting only *L* ^{*inter*}^ for Nyström method, we prevent from such loss. In addition, in contrast to various inversion algorithms for block tri-diagonal matrices, Nyström method is utilization of rectangular banded diagonal blocks combined with property of the graph Laplacian.

Finally, with respect to integrative analysis of multi-omics data, the overall complexity (13) is reduced from *O*(*N* ^3^), achieving faster matrix inversion. Since the size of multi-omics data can get very large, the proposed method can adjust effectively to multi-layer structure of omics.

## Experiments

### Data

To validate the performance of the proposed method, we compared the performance of the multi-layered network with the proposed method to that of the non-hierarchical single layered network with ordinary semi-supervised learning scheme. For problem setting, we applied it to disease co-occurrence prediction problem on two-layered network consisting of symptom-layer and disease-layer. Disease co-occurrence prediction has importance for treatment and prevention, in practice [46]. For example, examining disease co-occurrence of cancer, which has high disease co-occurrence rate, can serve as a crucial prognostic factor for patients with cancer [47] and has direct influence on treatment of patients [48]. Therefore, disease co-occurrence had been studied but only on single layer of omics [38]. In our study, we employ the fact that knowing common symptoms of two diseases can aid disease co-occurrence prediction. For instance, knowing that a patient has coughing can lead to a diagnosis of both flu and pneumonia, which are co-occurring diseases.

To construct the multi-layered network of symptoms and diseases, a list of disease and symptoms was obtained from Medical Subject Headings (MeSH) of the National Library of Medicine [49], yielding 4,318 diseases and 322 symptoms. For disease co-occurrence information, we collected the data from HuDiNe [50], which contained information for 1,015 diseases, out of 4,318 diseases. The obtained diseases were selected as nodes for disease-layer and 319 symptoms, out of 322 symptoms, with symptom-disease information from [17] were selected as nodes for symptom-layer. For intra-layer relations of diseases, *W* ^{*Disease*}^, we utilized similarity between diseases in terms of shared proteins (out of 15,777 proteins). For similarity measurement, we used Tanimoto kernel [51] which is given as

15 $$ {W}_{i j}=\frac{x_i\cdot {x}_j}{{\left\Vert {x}_i\right\Vert}^2+{\left\Vert {x}_j\right\Vert}^2-{x}_i\cdot {x}_j}, $$

where *x*_*i*_ and *x*_*j*_ are given as bit vectors. For intra-stratum relations of symptoms, *W* ^{*Symptom*}^, we utilized similarity between symptoms in terms of disease accompanying the symptoms. Tanimoto kernel was also used as similarity measurement for symptom relations. For inter-layer relations of symptom and disease, we used the symptom-disease data and binary weight where *W*_*ij*_^{*inter*}^ = 1, if co-occurrence is present, and *W*_*ij*_^{*inter*}^ = 0, otherwise, for *i* ∈ *Disease*, and *j* ∈ *Symptom*. Table 1 summarizes the data.

**Table 1:** Data source for symptom-disease stratified network and disease co-occurrence information

Data	Number of data	Sources
Symptom-Disease	319 symptoms/2,454 diseases	Supplementary information in [17]
Disease	4318 diseases/15,777 proteins	CTD, GAD, OMIM, PharmGKD, TTD
Disease Co-occurrence	1,015 diseases	HuDiNe

**Fig. 2:**
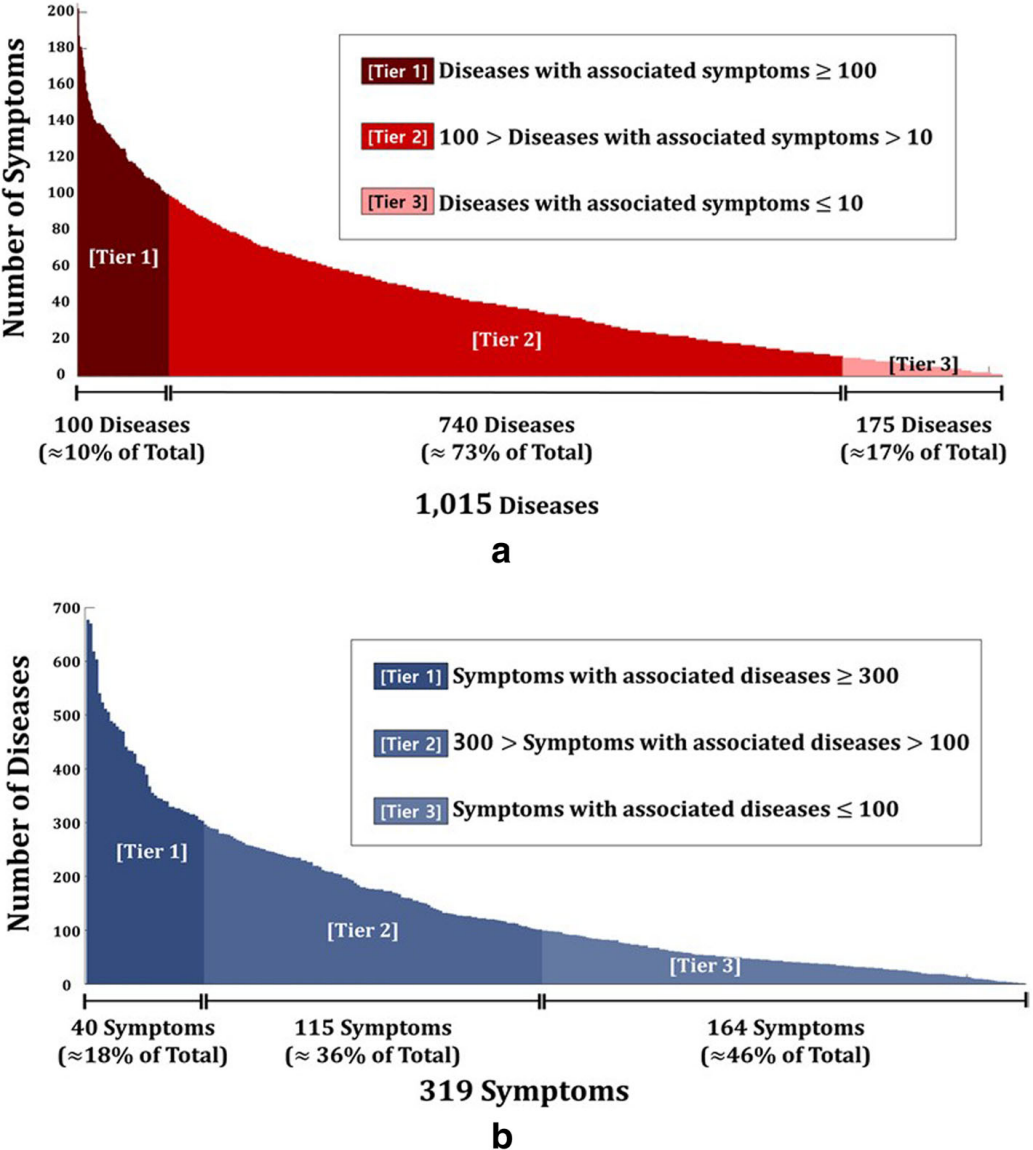
**a** Bar graph of the number of associated symptoms for a particular disease. **b** Bar graph of the number of associated disease for a particular symptom

Figure 2a shows the number of associated symptoms for a particular disease. Out of 1,015 diseases, brain neoplasm has the most number of associated symptoms with 202 records, followed by HIV infections, Lewy body disease, and cerebral hemorrhage. About 10% of diseases have 100 or more associated symptoms, about 73% have associated symptoms in between 100 and 10, and about 17% have less than or equal to 10 associated symptoms. Similarly, Fig. 2b show the number of associated diseases for a particular symptom. Out of 319 symptoms, pain is the most common symptom among diseases (677 diseases), followed by fever, change in body weight, and edema. About 18% of symptoms have 300 or more associated diseases, about 36% have associated diseases in between 300 and 100, and about 46% have less than or equal to 100 associated diseases.

### Experimental setting

For disease co-occurrence prediction problem, we employ the disease scoring setting, as in [38], where the semi-supervised learning algorithm provides the scores for disease. With the two-layered network of symptom and diseases, we first selected a target disease and gave label ‘1’ to target disease, indicating the presence of diseases. For other unlabeled diseases, we gave label ‘0’s. Then, we randomly gave label ‘1’s to 0 ~ 100% on 20% interval to related symptoms and gave ‘0’s to unrelated symptoms. The 0% of labeled symptoms represent the reference network, or the single disease network. We assume that we know 20% of co-occurring diseases in a priori, and therefore we randomly set and assign 20% of co-occurring diseases with label ‘1’s. Note that we can change the percentages but the effect is similar for both single-layered network and multi-layered network. The parameters, *μ*_*a*_ and *μ*_*b*_ were determined in the range {0.01, …, 100} and the performance of two-layered network of symptoms and diseases was compared to that of the reference network. The performance was measured by Area Under ROC Curve (AUC) [52], which compared prediction output *f* = (*f*_1_, *f*_2_, …, *f*_*n*_)^*T*^ with true labels. For validation, Leave-One-Out method [53] was used and the experiment was repeated 10 times.

## Results and Discussion

### Results on validity of the proposed algorithm

**Fig. 3:**
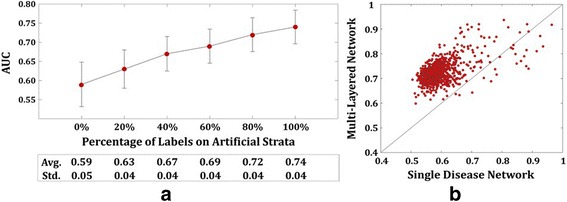
**a** Mean AUC for multi-layered network with 0 ~ 100%, on 20% interval, of labeled symptoms. 0% indicates the single disease network (reference network) where no labels and inter-stratum connections are given. **b** AUC for multi-layered network with 100% labeled symptoms against the reference network. Dots above diagonal line indicates higher AUC of multi-layered network for a particular target disease

The results are summarized in Fig. 3. Figure 3a illustrates AUC performance comparison in predicting disease co-occurrence for symptom-disease layered network and single disease network. It shows that for every increase in % of labels in symptom-layer achieves higher AUC than 0.59 of the reference network. Furthermore, it shows that increase in the number of labels for related symptoms leads to higher AUC performance. In the view of practitioner, this result suggests that knowing more symptoms disclose more information regarding characteristics of disease and its co-occurrence. Figure 3b depicts AUC for multi-layered network with 100% labeled symptoms against the reference network. If a point in scatter plot is above the diagonal line, then the multi-layered network performs better for a particular target disease. From the figure, we can see that most of the points are above the diagonal line, indicating better performance of the multi-layered network over the reference network. Such results consolidate the fact that labels in symptom-layer can benefit predictions for disease co-occurrence.

### Enrichment analysis: relevance of use of symptom data for disease co-occurrence

**Fig. 4:**
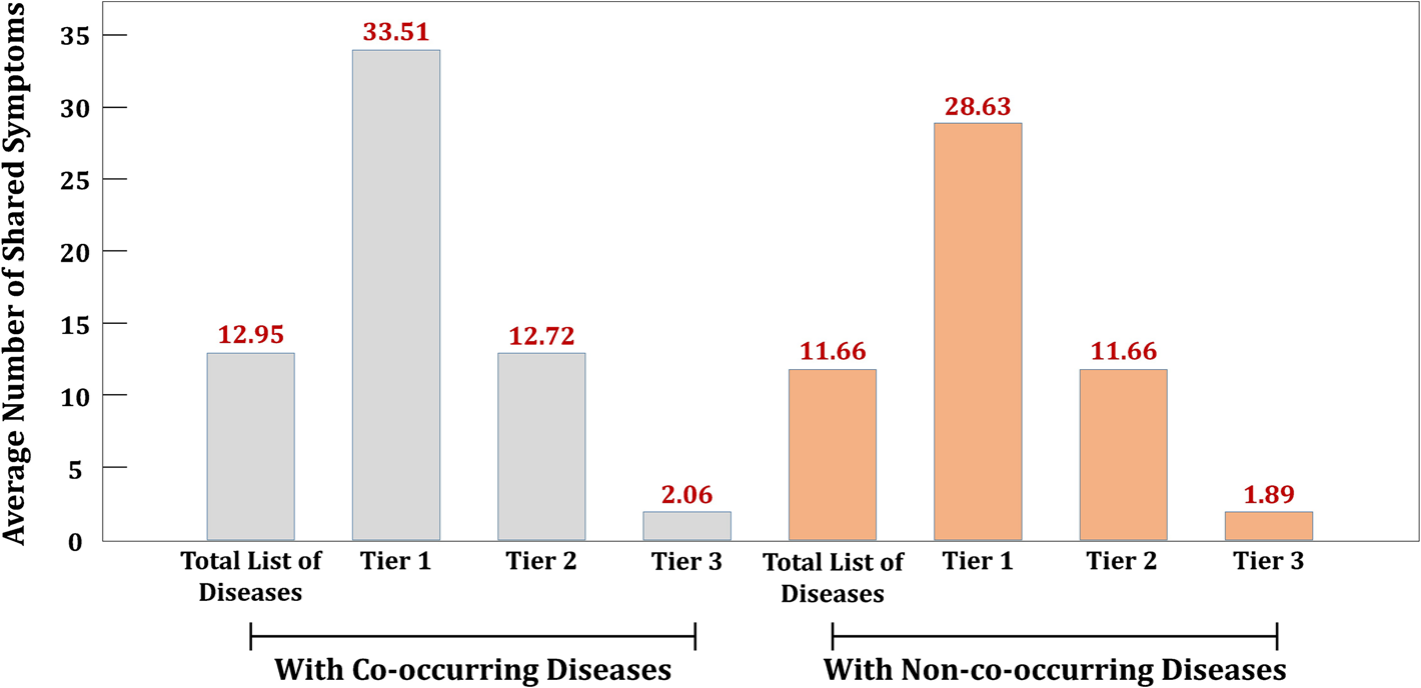
Comparison of the average number of shared symptoms with co-occurring diseases and with non-co-occurring diseases for total list of diseases, tier 1, tier 2, and tier 3

To examine relevance of use of symptoms for disease co-occurrence, we compared the difference between the average number of shared symptoms with co-occurring diseases and non-co-occurring diseases for each target disease. The main reason for such inspection is that the number of shared symptoms affect inter-layer label propagation in semi-supervised learning setting. If there exists a significant difference between the average number of shared symptoms with co-occurring diseases and non-co-occurring diseases for a target disease, then symptoms, indeed, have relevance with disease co-occurrence. Figure 4 illustrates the average number of shared symptoms with co-occurring and non-co-occurring diseases, respectively, for total list of diseases and the tiers that correspond to those in Fig. 2a. For statistical evaluation, we carried out one sided t-test of difference in means for each group, where the null hypothesis is that the difference in means is zero and alternative is that the average of shared symptoms with co-occurring diseases is higher than that with non-co-occurring diseases. The results are shown in Table 2.

**Table 2:** Results for statistical evaluation with one-sided t-test for difference in means

	Total list of diseases	Tier 1	Tier 2	Tier 3
*p*-value	<0.001	<0.001	<0.001	<0.001
T-statistics	11.238	5.558	12.131	6.391
Degree of Freedom	1,014	100	738	174
Standard Deviation	3.654	8.822	2.378	0.368

In Fig. 4, it shows that the average number of share symptoms is higher with co-occurring disease than that with non-co-occurring diseases for each group. It is also noticeable to see that in Table 2, the results of t-tests allow us to reject the null hypothesis for each case with *p*-value <0.001 and conclude the alternative. Thus, we can deduce that there exists a definite relevance between shared symptoms between diseases and disease co-occurrence.

**Fig. 5:**
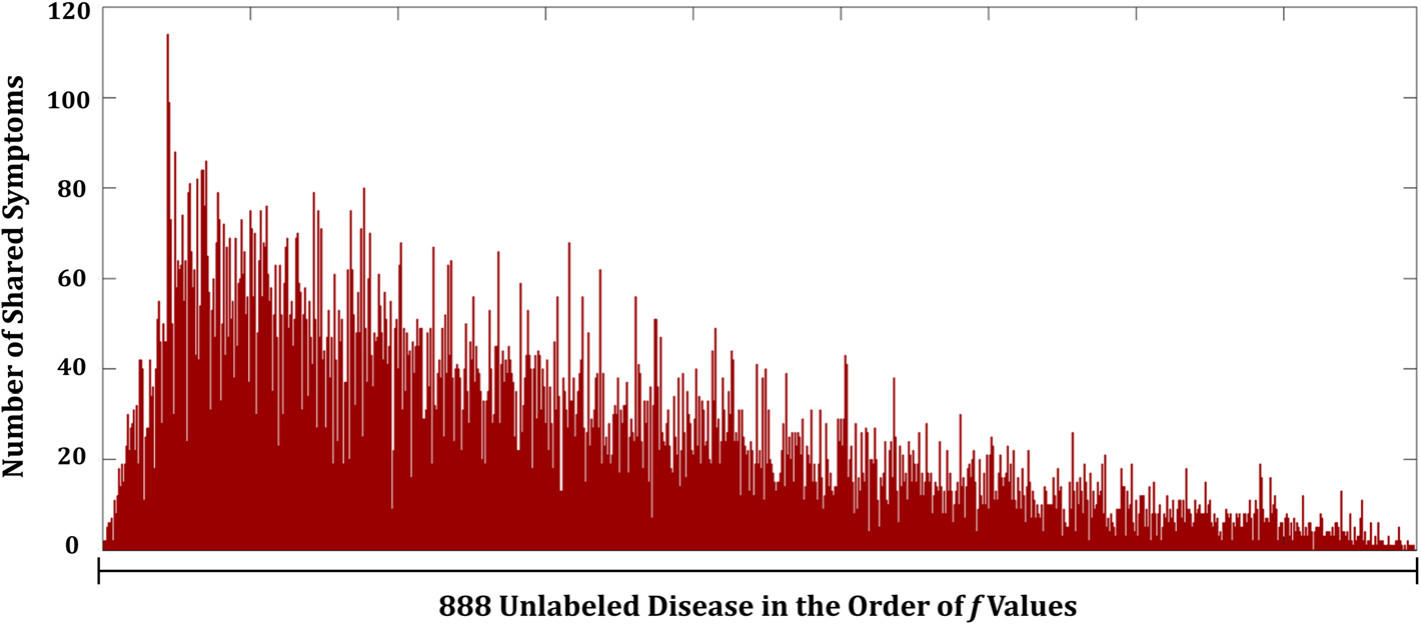
Number of shared symptoms for Thrombocytopenia in the order of *f* values

To elucidate more understanding of effects in use of symptom-layer, we selected thrombocytopenia as the target disease and analyzed the distribution of the number of shared symptoms. Thrombocytopenia refers to any disorders in which there is an abnormally low amount of platelets that help blood to clot [54, 55]. Figure 5 shows the number of shared symptoms with other diseases, in the order of value of predicative output, *f*, in eq. (12). These values represent relative closeness to being labeled as co-occurring disease with the target disease compared to one another. In Fig. 5, it shows that higher number of shared symptoms yields relatively higher value of predicative output of predicting disease co-occurrence. This solidifies the relevance of use of symptoms for prediction of disease co-occurrence.

## Conclusion

In this paper, we develop a graph based semi-supervised learning for prediction process in multi-layered biomedical systems. The algorithm involves matrix separation and graph integration methods but issues with computational complexity and sparseness must be solved. To resolve the issues, we devise a revised matrix inversion scheme consisting of Nyström method and Woodbury formula. Theoretically, the proposed method can reduce computational complexity by coping with sparseness, while preserving innate structure and properties of each layer.

To test the proposed algorithm, it was applied to two-layered system of symptoms and diseases to predict disease co-occurrence. The results showed improvement in prediction in terms of AUC where the performance increased from 0.59 of single disease network to 0.74 of symptom-disease network. Furthermore, it also showed relevance of use of symptoms on disease co-occurrence prediction with statistical evidence for higher average of shared symptoms with co-occurring diseases than that of non-co-occurring diseases. In theoretical perspective, although the proposed algorithm was applied on two-layered network for our experiments, it has scalability power as it is applicable to multi-layered structure with large number of biomedical data, and achieves faster inversion than normal matrix inversion.

As an extension of the research, since disease co-occurrence prediction problem has been studied for many years, it is possible to consider comparing the proposed method with other works such as [56]. In addition, we can consider extending additional layers where the extra layers convey relevant information. In case of disease co-occurrence prediction, inclusion of additional layers of phenotype/clinical data would be beneficial as they serve as important information to construct comorbidity map. In different perspective, we can also consider cases outside the box of the central dogma of biology, where multi-layered network can exist in a non-hierarchical structure.

On the other hand, the research has novelty in that it is a new integrative algorithm that incorporates vertical structure of omics data, on contrary to other existing methods that integrate the data in parallel fashion. Moreover, the experiment results not only reflect the viewpoints of practitioners where they observe or seek for symptoms as primary diagnosis but also provide enhanced guideline for disease co-occurrence prediction, where it has importance for treatment and prevention in practice. Thus, the proposed algorithm can serve as a valuable tool for inference process of multi-layered biological system.
